# Reconstructive surgery outreach to low- and middle-income countries: An interdisciplinary analysis of 131 non-governmental organizations

**DOI:** 10.7189/jogh.12.04002

**Published:** 2022-02-05

**Authors:** Albert H Chao, Jacqueline R McAllister

**Affiliations:** 1 Ohio State University, Department of Reconstructive Surgery, Columbus, Ohio, USA; 2 Kenyon College, Department of Political Science, Gambier, Ohio, USA

## Abstract

**Background:**

A significant portion of surgical aid to low- and middle-income countries (LMICs) is provided by non-governmental organizations (NGOs) in concert with surgeons, but little is known about the overall scope of this work or how it corresponds to indicators typically used to guide developmental aid distribution. The objective of this study was to characterize and investigate the collective efforts of NGOs providing reconstructive surgical aid to LMICs.

**Methods:**

An interdisciplinary approach was taken drawing from political science to examine this issue in reconstructive surgery. NGOs providing reconstructive surgical aid were identified, and then catalogued with respect to the LMICs they serve. LMICs were characterized using 28 variables in 6 domains based on contemporary developmental theory. Univariate and multivariate regression analyses were performed.

**Results:**

A total of 131 reconstructive surgery NGOs were identified serving 718 sites in 136 LMICs. Univariate analysis found that LMICs that were more frequent recipients of aid were more populous (*P* < 0.001), had lower ‘Hospital Beds Density’ (*P* = 0.001), and had higher rates of ‘Mortality by Injury’ (*P* = 0.001). Multivariate regression analysis identified population as the sole predictor among all indicators analyzed (95% confidence interval (CI) = 1.154 to 1.469; *P* = 0.001).

**Conclusions:**

The distribution of reconstructive surgical aid by NGOs is guided most by population, but not other characteristics traditionally used to guide aid distribution. Greater coordination and data-sharing among NGOs is recommended to optimize outreach efforts.

Approximately 30% of the global burden of human disease consists of conditions that are surgical [1]. Recent estimates indicate that 5 billion people lack access to safe, affordable surgical and anesthesia care when needed [2]. This results in reduced welfare for millions of people and stunts the development of low- and middle-income countries (LMICs). Accordingly, Dr Jim Yong Kim, former President of the World Bank, declared that “surgery is an indivisible, indispensable part of health care” and called for a “shared vision and strategy for global equity in essential surgical care” [3]. Non-governmental organizations (NGOs) and the surgeons who work with them play a crucial role in providing surgical aid to LMICs.

The fundamental goal of aid, whether by NGOs or governments, is development. Different schools of thought exist among contemporary developmental theorists regarding how this occurs [4]. Statists, for example, assert that the state is central and must actively promote essential areas, such as education and health services, in order to make the best use of developmental aid. Neoinstitutionalists, on the other hand, posit that the structure of institutions is more critical and that development is most successful when they safeguard fundamental rights such as equality, property, and the rule of law. Still others place an emphasis on human development and the diverse needs and interests of people, citing human capital as a key driver of development. A common theme amongst these views is that development depends on the context in which it is provided. Accordingly, numerous international organizations have devised and employed country-specific indicators that inform decision-making on how best to distribute developmental aid [5-7].

Little is known about the overall scope of NGOs that provide surgical aid to LMICs, or how these efforts correspond to LMIC indicators typically used to make assessments about developmental aid distribution. This topic lies at an intersection of medicine and political science, and thus we have sought to take an interdisciplinary approach to investigating this subject. Drawing from the field of political science, this study applies principles and methodology related to LMIC development to explore these issues in the field of reconstructive surgery. The specific objectives of this study are 2-fold. First, this study aims to identify NGOs that provide reconstructive surgical aid and to define the overall scope of their work with respect to the LMICs they serve. Second, we seek to characterize the relationship between the extent to which individual LMICs are recipients of reconstructive surgical aid by NGOs with country-specific indicators typically used to make assessments about developmental aid distribution. These indicators include those that relate to demography, economy, geography, government and society, health, and stability. This analysis thus aims to improve our understanding of the important work performed by NGOs and the surgeons that work with them, as well as to inform future outreach efforts.

## MATERIALS AND METHODS

### Non-governmental organizations

First, NGOs providing surgical aid to LMICs were identified by applying the methodology of Ng-Kamstra et al [8,9]. Briefly, their methodology defines a surgical NGO as one that fulfills two criteria. First, the organization meets the United Nations Rule of Law definition of a NGO, described as “a not-for-profit group, principally independent from government, which is organized on a local, national, or international level to address issues in support of a public good” [10]. Second, the NGO performs surgery in at least one LMIC, where surgery is defined as the “therapeutic excision, incision, or manipulation of tissue in an operating room, and distinguished from the logistical or financial support of such care” [8]. Based on this definition, surgical NGOs were identified through a review of internet NGO databases and resources [8,9]. In this study, in order to maximize identification of reconstructive surgical NGOs, we additionally reviewed the Plastic Surgery Foundation Volunteers in Plastic Surgery database [11].

Second, to identify surgical NGOs specifically providing reconstructive surgical aid, we then applied additional inclusion criteria for NGOs that perform reconstructive surgery. The list of LMICs served by each NGO was recorded, which typically represented their work over multi-year time periods given that many NGOs have rotating or variable service schedules. Non-governmental organizations were excluded if there was insufficient publicly available data to determine their areas of treatment and/or service sites.

### Variables

The dependent variable in this study was a country-specific variable of the total number of times a LMIC was listed as a site served by reconstructive surgery NGOs. In the statistical analysis, this was treated as a count variable for each LMIC. The independent variables consisted of 28 country-specific characteristics and indicators in 6 domains based on contemporary developmental theory. These country-specific characteristics are from the year 2020, except where otherwise noted. A summary of these variables is presented in **Table 1** with specific key details to follow (see Appendix S1 in the **Online Supplementary Document** for additional technical details).

**Table 1:** Independent variables characterizing low- and middle-income countries.

Category	Variable	Source
Demography	English-speaking	CIA [12]
	Internet use	CIA
	Literacy	CIA
	Population	CIA
	Urbanization	CIA
Economy	GDP per capita	World Bank [7]
	GDP healthcare	World Bank
	Healthcare dollars per capita	World Bank
	Income classification	World Bank
Geography	Airports	CIA
	Continent	Google [13]
	Distance	Google
Government & Society	Freedom	Freedom House [14]
	Human development index	UNDP [15]
	Polity score	Polity Project [16]
	Press freedom	Freedom House
Health	Healthy life expectancy at birth	World Bank
	Hospital beds density (per 1000)	World Bank
	Infant mortality (per 1000 live births)	WHO [17]
	Maternal mortality (per 100 000 live births)	World Bank
	Mortality by injury	World Bank
	Physicians density (per 1000)	World Bank
	Surgical specialist workforce (per 100 000)	Lancet [18]
Stability	Armed conflict	UCDP [19]
	Civil unrest	US Department of State [20]
	Crime	US Department of State
	Terrorism	US Department of State
	Travel advisory	US Department of State

CIA – Central Intelligence Agency, GDP – gross domestic product, IQR – interquartile range, UCDP – Uppsala Conflict Data Program, WHO – World Health Organization.

**Economy.** ‘Income Classification’ is a categorical variable (low, lower-middle, upper-middle) based on gross national income per capita [21]. This classification by the World Bank is a widely used metric for development due to its close correlation with other non-monetary measures of quality of life [22].

**Government and Society.** ‘Freedom’ is a categorical variable (Free, Partly Free, Not Free) based on 10 political rights and 15 civil liberties indicators [23]. ‘Press Freedom’ is a categorical variable (Free, Partly Free, Not Free), and is based on 23 measures of the legal, political, and economic environment for the media in a country [24].

The ‘Human Development Index’ is a summary measure of average achievement in key dimensions of human development: a long and healthy life, being knowledgeable, and having a decent standard of living [15]. It was created by the United Nations Development Programme on the view that people and their capabilities represent important criteria for assessing the development of a country, not economic growth alone.

‘Polity’ is a political science data set that reports a state’s level of democracy. Its conclusions are based on an evaluation of that state's elections (eg, whether they are competitive and open), the nature of political participation in general, and the extent of checks on executive authority [16].

**Healthcare.** ‘Healthy Life Expectancy at birth’ is a population health measure that combines mortality data with morbidity and health status data to estimate expected years of life in good health that a newborn can expect [25]. This measure is frequently used to estimate and predict future health service needs, evaluate health programs, and identify trends and inequalities [26]. ‘Mortality by Injury’ is a surrogate measure for the incidence and severity of trauma.

**Stability.** ‘Armed Conflict’, ‘Civil Unrest’, ‘Terrorism’ are binary variables indicating their presence or absence [19,27]. ‘Crime’ is a binary variable and indicates whether levels are low or high as classified by the US Department of State. ‘Travel Advisory Level’ is an ordinal variable indicating recommendations regarding travel (exercise normal precautions, reconsider travel, exercise increased caution, do not travel) [28].

### Statistical analysis

**Descriptive statistics.** Descriptive statistics were used to present the characteristics of LMICs as a group, as well as weighted by NGO representation. For example, if Country 1 has a population of A and was a recipient of aid by X NGOs, and Country 2 has a population of B and was a recipient of aid by Y NGOs, then the weighted mean population of countries receiving reconstructive surgical aid was calculated as equal to (A × X + B × Y) / (X + Y).

**Univariate.** Each independent variable was analyzed with respect to the dependent variable using the Mann-Whitney U test for binary variables, Kruskal-Wallis test for categorical variables, and Pearson correlation for continuous variables. All tests were two tailed. *P* values <0.05 were considered significant.

**Multivariate.** Regression analysis was employed to determine independent predictors for reconstructive surgical aid by NGOs to LMICs. Since the dependent variable was a count variable, both Poisson and negative binomial regression models were explored. Goodness of fit of the final model was determined using the Omnibus Test and Residuals Analysis of the difference between actual responses and the estimated values from the model.

All analyses were performed using SPSS version 27 (IBM Corp, Armonk, NY, USA).

## RESULTS

### Low- and middle-income countries

A total of 136 LMICs were included in this study, which includes all LMICs except one (Kosovo). Of these, 31 (22.6%) were low-income, 47 (34.3%) were lower middle-income, and 59 (43.1%) were upper middle-income nations. Overall LMIC characteristics and LMIC characteristics weighted by NGO representation are presented in **Table 2**.

**Table 2:** Characteristics of low- and middle-income countries and weighted by NGO representation

	LMICs (n = 137)	LMICs weighted by NGO representation (n = 718)
**Demography:**		
English-speaking	57 (41.6%)	511 (71.2%)
Internet access	31.5% (IQR, 19.1%-53.8%)	30.0% (IQR, 21.3%-54.1%)
Literacy	84.4% (IQR, 61.8%-96.7%)	78.7% (IQR, 62.3%-92.7%)
Population	10.2M (IQR, 2.4-29.0M)	28.1M (IQR, 10.8-95.5M)
Urbanization	52.1% (IQR, 36.7%-69.2%)	51.8% (IQR, 34.9%-63.3%)
**Economy:**		
GDP per capita	US$3360 (IQR, US$1370-6,289)	US$2549 (IQR, US$1482-6,267)
GDP healthcare	5.7% (IQR, 4.3%-7.0%)	5.5% (IQR, 4.2%-6.1%)
Healthcare dollars per capita	US$165 (IQR, US$62-344)	US$129 (IQR, US$55-316)
**Income classification:**		
Low	31 (22.6%)	162 (22.6%)
Lower-middle	47 (34.3%)	301 (41.9%)
Upper-middle	59 (43.1%)	255 (35.5%)
**Geography:**		
Airports	11 (IQR, 4-30)	16 (IQR, 7.75-72)
Continent:		
Africa	54 (39.4%)	218 (30.4%)
Asia	30 (21.9%)	232 (32.3%)
Europe	12 (8.8%)	22 (3.1%)
North America	15 (10.9%)	129 (18.0%)
South America	26 (19.0%)	117 (16.3%)
Distance	10 476 km (IQR, 8515-12,714)	10 969 km (IQR, 5576-13,219)
**Government & society:**		
Freedom:		
Not free	44 (32.1%)	198 (27.6%)
Partially free	55 (40.1%)	356 (49.6%)
Free	37 (27.0%)	160 (22.3%)
Human Development Index	0.6685 (IQR, 0.5375-0.7463)	0.6490 (IQR, 0.5600-0.7313)
Polity score	4.0 (IQR, -1.5-8.0)	5.0 (IQR, 0.0-8.0)
Press freedom:		
Not free	59 (43.1%)	299 (41.6%)
Partially free	57 (41.6%)	367 (51.1%)
Free	21 (15.3%)	51 (7.1%)
**Health:**		
Healthy life expectancy at birth	55.5 (IQR, 45.0-61.0)	55.0 (IQR, 44.75-61.0)
Hospital beds’ density	1.8 (IQR, 1.0-3.3)	1.2 (IQR, 0.80-1.96)
Infant mortality	22.5 (IQR, 13.2-39.3)	22.5 (IQR, 13.6-35.5)
Maternal mortality	114.5 (IQR, 36.0-316.3)	123.0 (IQR, 59.0-308.0)
Mortality by injury	8.9 (IQR, 7.4-10.9)	10.1 (IQR, 8.0-11.9)
Physicians’ density	0.8 (IQR, 0.2-1.9)	0.8 (IQR, 0.2-1.7)
Surgical specialist workforce	7.6 (IQR, 1.4-30.3)	5.1 (IQR, 1.0-22.2)
Stability:		
Armed conflict	33 (24.1%)	205 (28.6%)
Civil unrest	34 (24.8%)	247 (34.4%)
Crime	50 (36.5%)	268 (37.3%)
Terrorism	40 (29.2%)	252 (35.1%)
**Travel advisory:**		
1 (Exercise normal precautions)	57 (41.6%)	175 (24.4%)
2 (Exercise increased caution)	54 (39.4%)	399 (55.6%)
3 (Reconsider travel)	12 (8.8%)	86 (12.0%)
4 (Do not travel)	14 (10.2%)	58 (8.1%)

IQR – interquartile range, LMIC – low- and middle-income countries, NGO – non-governmental organization

### Non-governmental organizations

**Figure 1:**
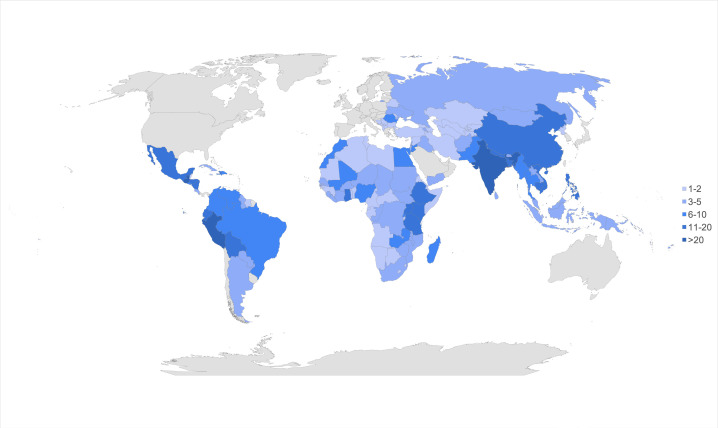
Collective worldwide efforts of NGOs in terms of the number of NGOs that provide reconstructive surgery aid to individual LMICs.

A total of 131 NGOs met inclusion criteria as NGOs that provide reconstructive surgical aid. Collectively, a total of 718 sites in 136 LMICs were served by these NGOs, of which 162 (22.6%) were in low-income, 301 (41.9%) in lower middle-income, and 255 (35.5%) in upper middle-income countries. The distribution of work performed by these NGOs by continent was Africa (30.4%), Asia (32.3%), Europe (3.1%), North America (18.0%), and South America (16.3%). The collective worldwide efforts of these NGOs are depicted in **Figure 1**.

### Univariate analysis

The results of univariate analysis are presented in **Table 3**. Countries with larger populations were more frequent recipients of reconstructive surgical aid by NGOs (Pearson = 0.52, *P* < 0.001), as were countries with more airports (Pearson = 0.29, *P* = 0.001) and those located in Africa and Asia (*P* = 0.008). Receipt of aid was greater in the middle-income than in low-income groups (*P* = 0.043). Partially free and unfree LMICs were more often visited by NGOs than free LMICs with respect to both overall and press freedom (*P* = 0.012 and *P* = 0.011, respectively).

**Table 3:** Univariate analysis of country-specific variables on receipt of reconstructive surgical aid to LMICs by NGOs

	*P*-value
**Demography:**	
English-speaking	0.91
Internet access	0.88
Literacy	0.79
Population	<0.001*
Urbanization	0.50
**Economy:**	
GDP per capita	0.27
GDP spent on health care	0.18
Healthcare dollars per capita	0.30
Income classification	0.043*
**Geography**	
Airports	0.001*
Continent	0.008*
Distance	0.17
**Government & society:**	
Freedom	0.012*
Human Development Index	0.83
Polity score	0.10
Press freedom	0.011*
**Health:**	
Healthy life expectancy at birth	0.66
Hospital beds’ density	0.001*
Infant mortality	0.41
Maternal mortality	0.62
Mortality by Injury	0.009*
Physicians’ density	0.37
Surgical specialist workforce	0.28
**Stability:**	
Armed conflict	0.46
Civil unrest	0.014*
Crime	<0.001*
Terrorism	0.25
Travel advisory	0.003*

GDP – gross domestic product, LMIC – low- and middle-income countries, NGO – non-governmental organization

*P < 0.05.

Countries with lower ‘Hospital Beds Density’ were more frequent recipients of reconstructive surgical aid by NGOs (Pearson = -0.28, *P* = 0.001), while no correlation was identified with respect to ‘Physicians Density’ (*P* = 0.37) or ‘Surgical Specialist Workforce’ (*P* = 0.28). In addition, LMICs with higher rates of ‘Mortality by Injury’ were more frequently represented (Pearson = 0.23, *P* = 0.009). Countries with ongoing civil unrest (*P* = 0.014), high crime rates (*P* < 0.001), or a travel advisory (*P* = 0.003) were more frequently served by NGOs.

### Multivariate analysis

Regression analysis was performed to delineate the relationship between the extent to which individual LMICs were recipients of reconstructive surgical aid and the characteristics of those LMICs. The mean and variance of the data were dissimilar (ie, overdispersion), and thus a negative binomial regression model was selected. The Omnibus Test indicated a statistically significant overall model (*P* < 0.001). In addition, Residuals Analysis demonstrated that all residuals ranged from -2.0 and +2.0, additionally confirming good model fit. There was one statistically significant independent predictor of NGO activity: ‘Population’ (95% confidence interval (CI) = 1.154 to 1.469; *P* = 0.001).

## DISCUSSION

Compared to governments, NGOs possess a number of characteristics that make them duly suited to providing health care in resource-restricted settings [29]. They operate with few restrictions, allowing them to better adapt to local conditions and respond to specific health care needs. For example, NGOs are not required to notify government agencies about their activities, membership, or outreach. There are also few legal restrictions on the freedoms of expression and association of NGOs [30]. In addition, NGOs have the ability to communicate at all levels within a given setting, ranging from neighborhoods to the top levels of government. Lastly, NGOs are less subject to certain pressures such as geopolitical interests, which might interfere with project aims [31-33].

In this study, an interdisciplinary approach was undertaken to examine global outreach in reconstructive surgery using an original data set based on established methodology. A key finding was that in the multivariate analysis, only ‘Population’ was identified as an independent predictor of the frequency by which sites were served by NGOs. This is perhaps not a surprising result. Of the data that is available to organizations, population is one of the most consistently available and easiest to interpret. Certainly, larger countries have more people who might benefit from aid. However, decisions about the distribution of aid should also include a measure of need, such as the World Bank income classification. For example, China is an upper middle-income country that ranked eighth overall for the number of sites receiving of reconstructive surgical aid, surpassing 129 other countries with greater need. This issue is important in relation to *poverty trap* theory, a major concept in the discourse about how best to distribute developmental aid, and which concerns the self-reinforcing mechanisms that cause poverty to persist unless there is outside intervention [34]. For example, a joint report by the World Health Organization and the World Bank stated that approximately 100 million people annually are pushed into extreme poverty because they had to pay for health services out of their own pockets, causing countries that are already amongst the most poor to be at greatest risk for continued or worsening poverty [35].

From an interdisciplinary perspective, we suggest the following policy recommendations. First, NGOs providing reconstructive surgical aid should formally coordinate their efforts, which could potentially help reduce disparities in aid distribution. Currently, no such coordination mechanism or shared information warehouse exists. As early as the 1960s, organizations such as the United Nations (UN), Red Cross, and governmental and nongovernmental agencies recognized the importance of coordination. Accordingly, they proposed mechanisms and frameworks for coordination [36,37]. Much of this work originated in the context of delivering health services during humanitarian crises, but many of the same principles apply to health services generally: identify needs, assess infrastructure, inventory resources, and apply experience. The need for greater collaboration among NGOs in health care is not unique and is also well recognized in other areas of aid [38,39].

Lotfi et al. performed a systematic review of five suggested models for coordinating the provision of health services in LMICs when multiple actors are involved [40]. Of these, the “Who is Where, When, doing What” (4Ws) tool, created by the U.N. Inter-Agency Standing Committee, is readily applicable to surgical aid by NGOs [41]. As its name implies, the 4Ws tool focuses on who the participating actors are, where and when they are acting, and what their activities are. The 4Ws tool includes a one-page introduction and three activity spreadsheets: one for information about the organization, one for details of activities (eg, specific planned surgical procedures), and the last one for pre-defined activities (eg, engaging the local community). Exercises are then conducted with all involved parties aimed at mapping activities, recommending changes based on field experience, and presenting the findings to a coordination group. This tool was effective in coordinating responsibilities between health services actors responding to the Iraqi refugee crisis in Jordan following the Iraq War in 2003. Similar approaches for collaboration between NGOs have also been undertaken in the setting of surgery in Rwanda and primary health care in Pakistan [42,43].

A second policy recommendation is for NGOs to systematically study and report the outcomes associated with their work. Metrics of “Aid Effectiveness” are routinely measured and reported by international organizations such as the World Bank and the United Nations Educational, Scientific and Cultural Organization in all areas of development, and are crucial for understanding the effects of aid [44]. The Lancet Commission on Global Surgery has largely led the way in the field of global surgery with measures such as ‘Access to Timely Essential Surgery’ [18]. These are critical indicators that speak broadly as to the surgical environment in LMICs. However, additional information is needed about the factors that underlie these measures. This is where more could be done by NGOs. While it is likely that NGOs each perform their own internal analyses, a shared approach to data would do much toward advancing the mission of global surgery. Even basic information, such as the volume of key procedures performed in a LMIC before and after outreach, would benefit all stakeholders and help to maximize the impact of aid efforts. Indeed, many experts have advocated greater inclusion of surgical measures like these in the WHO’s Core Health Indicators [45,46].

This study has several limitations. First, relevant data about LMICs is sometimes limited, including variables that were of potential interest in this study. For example, we had planned to include the World Bank’s ‘Number of Surgical Procedures (per 100 000 population)’ as a variable in the analysis. However, this was precluded due to inconsistent availability of this data for LMICs, which is an example of the broader challenge of obtaining complete and accurate data about LMICs. Another important category of data that was inconsistently available was information about the incidences of conditions treated by reconstructive surgeons (eg, cleft lip/palate) in individual LMICs. Second, it is not possible to confirm whether every existing and relevant reconstructive surgery NGO was captured in this study. This is due in large part to the loose and broad definition of NGOs. While this characteristic represents a strength of NGOs with respect to the ease with which they may assemble and act, it also means that publicly available information may be limited. Furthermore, our methodology may not have captured joint trips or trips involving multiple surgical specialties. Nevertheless, we believe that the analysis of a large number of NGOs in this study may help improve our understanding of this subject. Future work should aim to systematize and share the collection and analysis of data on the work performed by NGOs, in both reconstructive surgery and other surgical subspecialties, to better understand why they work where they do and their impact.

## CONCLUSIONS

The scope of countries served by NGOs that provide reconstructive surgical aid is broad, and is especially concentrated in more populous nations. We recommend greater coordination of efforts and data-sharing by NGOs in order to better match aid distribution with need. In addition, similar investigations in other surgical subspecialties may be advisable to help maximize the impact of the important work performed by NGOs and the surgeons with whom they work.

## Additional material


Online Supplementary Document
