# Supplementary material for: Reconstructive surgery outreach to low- and middle-income countries: An interdisciplinary analysis of 131 non-governmental organizations
Source: J Glob Health. 2022 Feb 5;12:04002. doi: 10.7189/jogh.12.04002 (PMC8822145; doi:10.7189/jogh.12.04002)
Supplement: Online Supplementary Document [file jogh-12-04002-s001.pdf]

Appendix S1. Details of independent variables characterizing low- and middle-income countries.

**Category**	**Variable**	**Source**	**Variable Type**	**Details**
Demography	English-speaking	CIA [11]	Nominal	Whether English is an official or second language or not
	Internet Use	CIA	Ratio	Percentage of population with internet access relative to entire population
	Literacy	CIA	Ratio	Percentage of population that is literate relative to entire population
	Population	CIA	Ratio	
	Urbanization	CIA	Ratio	Percentage of population that is urban relative to entire population
Economy	GDP per capita	World Bank [7]	Ratio	The total value of all the goods and services produced by a country in a particular year,

				divided by the number of people living there
	GDP Healthcare	World Bank	Ratio	Percentage of a country's GDP spent on health care
	Healthcare Dollars per capita	World Bank	Ratio	The amount that each country spends on health for both individual and collective services, divided by the number of people living there
	Income Classification	World Bank	Ordinal (low-income, lower middle-income, upper middle-income)	Low-income countries have a Gross National Income (GNI) per capita of $1,025 or less, lower middle-income countries between $1,026 and $3,995, and upper middle-income between $3,996 and $12,375
Geography	Airports	CIA	Ratio	Number of airports with paved runways
	Continent	Google [12]	Nominal (Africa,	

			Antarctica, Asia, Australia, Europe, North America, South America)	
	Distance	Google	Ratio	Kilometers calculated from centermost points of the United States and each LMIC
Government & Society	Freedom	Freedom House [13]	Ordinal (not free, partly free, free)	Based on 10 political rights and 15 civil liberties indicators
	Human Development Index	UNDP [14]	Ratio (0 to 1)	Summary measure of average achievement in key dimensions of human development: a long and healthy life, being knowledgeable, and having a decent standard of living

	Polity Score	Polity Project [15]	Ordinal (-10 to +10)	A state's level of democracy based on an evaluation of that state's elections, the nature of political participation in general, and the extent of checks on executive authority. (-10 to -6 corresponds to autocracies, -5 to 5 to anocracies, and 6 to 10 to democracies)
	Press Freedom	Freedom House	Ordinal (not free, partly free, free)	Based on 23 measures of the legal, political, and economic environment for the media in a country
Health	Healthy Life Expectancy at birth	World Bank	Interval	Population health measure that combines mortality data with morbidity and health status data to estimate expected years of life in good health that a newborn can expect
	Hospital Beds Density (per 1,000 population)	World Bank	Ratio	

	Infant Mortality (per 1,000 live births)	WHO [16]	Ratio	
	Maternal Mortality (per 100,000 live births)	World Bank	Ratio	
	Mortality by Injury	World Bank	Ratio	Percentage of deaths by injury relative to all deaths, distinguished from causes of death from communicable or noncommunicable diseases
	Physicians Density (per 1,000 population)	World Bank	Ratio	
	Surgical Specialist Workforce (per 100,000 population)	Lancet [17]	Ratio	Number of specialist surgical, anesthetic, and obstetric providers
Stability	Armed Conflict	UCDP [18]	Nominal	Contested incompatibility that concerns

			(present, absent)	government and/or territory where the use of armed force between two parties, of which at least one is the government of a state, results in at least 25 battle-related deaths in one calendar year
	Civil Unrest	U.S. Department of State [19]	Nominal (present, absent)	Political, economic, religious, and/or ethnic instability that may result in violence, major disruptions, or safety risks
	Crime	U.S. Department of State	Nominal (low, high)	Violent or organized crime
	Terrorism	U.S. Department of State	Nominal (present, absent)	Terrorist attacks have occurred and/or specific threats against civilians, groups, or other targets may exist

	Travel Advisory	U.S. Department of State	Ordinal (do not travel, reconsider travel, exercise increased caution, exercise normal precautions)	

CIA, Central Intelligence Agency; GDP, gross domestic product; IQR, interquartile range; UCDP, Uppsala Conflict Data Program; WHO, World Health Organization.
